# Surgical innovation in ophthalmology: challenges and opportunities

**DOI:** 10.1038/s41433-022-02324-8

**Published:** 2022-12-07

**Authors:** Augusto Azuara-Blanco, Aaron Carlisle, Hari Jayaram, Gus Gazzard, Daniel F. P. Larkin, Louisa Wickham, Noemi Lois, Amanda Bicket

**Affiliations:** 1 Centre for Public Health, Queen’s University Belfast, Belfast, UK; 2 Belfast Health and Social Care Trust, Belfast, UK; 3 NIHR Biomedical Research Centre & Glaucoma Service at Moorfields Eye Hospital NHS Foundation Trust, London, UK; 4 Institute of Ophthalmology, University College London, London, UK; 5 Cornea & External Diseases Service, Moorfields Eye Hospital, London, UK; 6 Vitreo-retinal Service, Moorfields Eye Hospital, London, UK; 7 Wellcome-Wolfson Institute for Experimental Medicine, Queen’s University, Belfast, UK; 8 Kellogg Eye Center, University of Michigan, Ann Arbor, MI USA

**Keywords:** Outcomes research, Health services

Ophthalmology is a high volume and rapidly evolving surgical specialty. Over the past few decades innovations in eye surgery have improved outcomes and safety, however novel surgeries can be associated with risks and require proper governance, evaluation and consent.

When considering the design, evaluation or adoption of a novel surgical technique ophthalmologists need to weigh up several factors, including the complexities associated with a surgical intervention where human factors can influence outcomes such as the surgeon’s expertise and learning curve. The conception of an original idea requires innovative thinking, whereas the development and evaluation of an idea requires methodical planning [1, 2], all underpinned by adequate governance and oversight.

The Royal College of Surgeons of England has issued guidelines on how novel surgeries should be introduced in clinical practice [3] and is promoting the use of the Idea/Innovation, Development, Exploration, Assessment, Long-term follow-up (IDEAL) Framework that offers a structured approach to surgical innovation [2]. We would propose that a similar process is adopted by the Royal College of Ophthalmologists.

## IDEAL phases

Typically, stage 1 is prompted by the need for a novel solution to a clinical problem and generates a case report or a short case series. This should include a clear and comprehensive explanation of the technique or equipment, including patient safety monitoring measures, pre- and post-procedure care and any adverse events experienced by the patient. Based on the outcomes of Phase 1, it will be possible to assess whether it is desirable to proceed with further patients and to address potential risks and take precautions [2].

Phase 2a results will determine if the technique and outcomes have reached stability in the hands of the current team and if the approach is ready for evaluation in a prospective, multi-centre IDEAL phase 2b cohort study. This should include criteria for patient selection, descriptions of eligible patients selected, an appreciation for an individual surgeon’s learning curve, a detailed explanation of the technique, including variants, and clinically important results and complications.

Phase 2b will provide the interpretation of data and evaluation of the feasibility of proceeding to RCT. In this regard, consensus should have been obtained on:

- Standardised technique (including accepted variants) and quality standards based on experience.
- Target patient population and indications.
- Outcome measure(s) (including estimated power calculation of the primary outcome).
- Comparator treatment for a trial.
- Willingness among operators and patients to accept randomisation [2].

The goal of stage 3 is to compare the efficacy between the conventional and novel interventions. Stage 4 focuses on long-term evaluation to assess innovation for “rare and long-term outcomes, and for variations in outcome” [3]. This should ideally be performed using prospective databases and registries with high user coverage.

## Training on novel surgeries

Ideally a surgical mentor with expertise in the novel method should supervise a certain number to establish competence for the trainee. This may vary depending on the mentee’s experience and skill. An organised training programme could help establish proficiency in the novel method.

Patients may be exposed to an increased risk of complications during the learning curve period in which a surgeon develops their skill in a new technique. The surgeon’s expertise with a novel technique should play a significant role in the informed consent process. However, this presents an ethical dilemma because it is difficult to disclose the risks of the learning curve to patients when those risks are unknown. An operator’s lack of self-awareness of their location on the learning curve may hinder their capacity to communicate risks to patients. Similarly, a patient’s wish to be considered for innovative techniques when no currently accepted treatment is available may leave them vulnerable to accepting higher levels of uncertainty and/or risk.

## Safety consent

The surgeon must facilitate the consent process by tailoring the discussion to the individual patient to ensure that they are aware of any risks that are significant to them as well as alternative treatment options. When a novel technology is involved, the consent process may be complicated, especially if there is a limited understanding of the potential risks and benefits during the early phases of development. If specific outcome data are available, surgeons should provide patients with this information. Char et al. found that 80% of patients responded that they could not consent to surgery if they did not know whether the surgeon would be doing the procedure for the first time [4].

Broekman et al. proposed that the following information [5] should be presented to patients:

- the innovative nature of the procedure;
- the surgeon’s experience with the new technique;
- the risks and benefits of the procedure, including possible unforeseeable or unknown risks or outcomes due to the “experimental and unvalidated nature of the procedure”;
- the evidence (or lack thereof);
- alternatives to the innovative procedure.

## Clinical governance

Surgical techniques that deviate significantly from standard practice must be underpinned by rigorous clinical governance. It is recommended that a surgical innovation committee be established to oversee the surgical innovation evaluation. The committee is responsible for guiding and protecting both surgeons and patients throughout the surgical innovation process.

For purposes of accountability, research integrity, and clinical governance, all patient-involved clinical trials should be recorded in databases available to the public worldwide.

Registration facilitates adherence to universally recognised ethical standards and precludes the alteration of primary outcomes based on findings during intermediate studies.

## Conclusion

Implementing the recommendations of the IDEAL partnership is an excellent next step for evaluating and improving the quality of surgical advances in ophthalmology.

## References

[1] Avery KNL, Wilson N, Macefield R, McNair A, Hoffmann C, Blazeby JM, et al. A core outcome set for seamless, standardized evaluation of innovative surgical procedures and devices (COHESIVE): a patient and professional stakeholder consensus study. Ann Surg. 2021. Epub ahead of print. 10.1097/SLA.0000000000004975 PMC9831031 34102667

[2] Hirst A, Agha RA, Rosin D, McCulloch P (2013). How can we improve surgical research and innovation?: the IDEAL framework for action. Int J Surg.

[3] Royal College of Surgeons Professional Standards Committee. Surgical innovation, new techniques and technologies—a guide to good practice. London: Royal College of Surgeons, England; 2019. https://www.rcseng.ac.uk/-/media/files/rcs/standards-and-research/standards-and-policy/good-practice-guides/old-documents/surgical-innovation-new-techniques-and-technologies.pdf.

[4] Lee Char SJ, Hills NK, Lo B, Kirkwood KS (2013). Informed consent for innovative surgery: a survey of patients and surgeons. Surgery.

[5] Broekman MLD. Ethics of innovation in neurosurgery. Cham, Switzerland: Springer Nature Switzerland AG; 2019.

[6] Angelos P (2010). The ethical challenges of surgical innovation for patient care. Lancet.

